# A236 ENDOSCOPIC MANAGEMENT OF RARE JEJUNAL SOLITARY PEUTZ-JEGHERS-TYPE POLYP IN AN ELDERLY PATIENT WITHOUT A POLYPOSIS SYNDROME: A CASE REPORT AND REVIEW OF THE LITERATURE

**DOI:** 10.1093/jcag/gwac036.236

**Published:** 2023-03-07

**Authors:** T Alenezi, T Bessissow, V Marcus

**Affiliations:** 1 Gastroenterology and Hepatology; 2 Pathology, McGill University Health centre, Montreal, Canada

## Abstract

**Background:**

Solitary Peutz-Jeghers-type polyp (SPJP) is a rare hamartomatous lesion. It is considered as a different entity from Peutz-Jeghers syndrome despite similar histopathological findings. It can be found in the GI tract but rarely in the jejunum. Jejunal SPJP are susceptible to necrosis, ulceration, and intussusception, resulting in GI bleeding or small bowel obstruction.

**Purpose:**

We describe a case of subacute gastrointestinal bleeding secondary to jejunal SPJP to share our approach to this challenging case using therapeutic endoscopy

**Method:**

An 81-year-old male patient with a history of atrial fibrillation on warfarin with stable therapeutic INR levels, who presented with 1-week history of melena, generalized fatigue and shortness of breath on exertion and was found to have profound iron deficiency anemia. Esophageal gastroduodenoscopy and colonoscopy failed to identify the source of bleeding; however, double balloon enteroscopy detected a 4 cm polyp with stalk in the proximal jejunum. Endoscopic polypectomy was performed and the whole polyp was removed. Histopathological examination was consistent with Peutz-Jeghers polyp. The oropharyngeal and skin examination was negative for mucocutaneous hyperpigmented macules and the genetic analysis was negative for STK11 mutation. Follow up magnetic resonance enterography and video capsule endoscopy did not reveal any other polypoid lesion in the GI tract. The patient’s symptoms resolved gradually, and his hemoglobin level returned back to normal levels within 6 months.

**Result(s):**

To our knowledge this is the first case of endoscopic polypectomy during balloon-assisted enteroscopy for jejunal SPJP.

**Conclusion(s):**

**Conclusion**

Solitary Peutz-Jeghers hamartomas are a rare occurrence but should be part of the differential diagnosis for abdominal pain from intussusception, or small bowel bleeding even in elderly patient. Balloon assisted enteroscopy is a valuable technique for the management of solitary small bowel lesions.

**Please acknowledge all funding agencies by checking the applicable boxes below:**

None

**Disclosure of Interest:**

None Declared

LIVER AND BILIARY DISEASE

